# The association between chiropractors’ view of practice and patient encounter-level characteristics in Ontario, Canada: a cross-sectional study

**DOI:** 10.1186/s12998-021-00398-x

**Published:** 2021-09-28

**Authors:** Jessica J. Wong, Sheilah Hogg-Johnson, André E. Bussières, Simon D. French, Silvano A. Mior

**Affiliations:** 1 Institute for Disability and Rehabilitation Research, Ontario Tech University and Canadian Memorial Chiropractic College, 2000 Simcoe Street North, Oshawa, ON L1G 0C5 Canada; 2 Dalla Lana School of Public Health, University of Toronto, 155 College Street, 6th Floor, Toronto, ON M5T 3M7 Canada; 3 Graduate Studies, Canadian Memorial Chiropractic College, 6100 Leslie Street, Toronto, ON M2H 3J1 Canada; 4 Department of Research and Innovation, Canadian Memorial Chiropractic College, 6100 Leslie Street, Toronto, ON M2H 3J1 Canada; 5 School of Physical and Occupational Therapy, Faculty of Medicine and Health Sciences, McGill University, 3654 Prom Sir-William-Osler, Montreal, QC H3G 1Y5 Canada; 6 Département Chiropratique, Université du Québec à Trois-Rivières, 3351 Boulevard des Forges, Trois-Rivières, QC G8Z 4M3 Canada; 7 Department of Chiropractic, Faculty of Medicine, Health and Human Sciences, Macquarie University, Level 3, 17 Wally’s Walk, North Ryde, NSW 2019 Australia

**Keywords:** Chiropractic, View of practice, Patient characteristics, Treatment characteristics, Cross-sectional study

## Abstract

**Background:**

Chiropractors have diverse views of practice, but the impact on their patient profiles and treatment approaches remains unclear. We assessed the association between chiropractors’ view of practice (unorthodox versus orthodox) and patient encounter-level characteristics among chiropractors who practice in Ontario, Canada.

**Methods:**

We conducted a cross-sectional study using Ontario Chiropractic Observation and Analysis STudy (O-COAST) data. In O-COAST, Ontario chiropractors were randomly recruited from a list of registered chiropractors in 2015 and recorded up to 100 consecutive patient encounters. We classified chiropractors’ response regarding their views of practice as unorthodox when viewing “vertebral subluxation as an encumbrance to health that is corrected to benefit overall well-being”; other views were considered orthodox. Patient encounter-level characteristics included: (1) non-musculoskeletal reason-for-encounter; (2) subluxation as diagnosis; (3) duration of encounter (log-transformed for modeling); (4) unimodal manipulative treatment; and (5) patient health characteristics (good health status, some activity limitations). We conducted multilevel logistic regression to assess the association between view of practice and aforementioned characteristics, accounting for potential confounders and clustering of encounters within chiropractors. The multilevel models had two levels (level 1—patient encounter level; level 2—chiropractor level), with level 1 patient encounters nested within level 2 chiropractors.

**Results:**

We included 40 chiropractors (mean age = 43.4 years, SD = 11.5) and 3,378 chiropractor-patient encounters. The 2,332 unique patients identified had a mean age of 48.5 years (SD = 18.5). Chiropractors with unorthodox views had higher odds of having patients with a non-musculoskeletal reason-for-encounter (adjusted odds ratio (aOR) 16.5, 95% CI 3.2–84.0) and subluxation as diagnosis (aOR 63.0, 95% CI 4.2–949.1). Encounters of chiropractors with unorthodox views were 0.6 times shorter than those with orthodox views (95% CI 0.4–0.9). Chiropractor level explained 32%, 75%, and 49% of the variability in non-musculoskeletal reason-for-encounter, subluxation as diagnosis, and encounter duration, respectively. We observed no association between unorthodox view and unimodal manipulative treatment or patient health characteristics.

**Conclusions:**

Chiropractors’ unorthodox view of practice was associated with treating non-musculoskeletal conditions, subluxation as diagnosis, and shorter duration of encounter. Chiropractor level explained a high proportion of variability in these outcomes. Findings have implications for understanding chiropractic practice and informing interprofessional collaboration.

**Supplementary Information:**

The online version contains supplementary material available at 10.1186/s12998-021-00398-x.

## Background

Chiropractic is a healthcare profession concerned with the management of musculoskeletal conditions, and a commonly used complementary and alternative medicine therapy [1–3]. Chiropractors practice in more than 100 countries worldwide, with the largest distribution in the United States and Canada [4]. A recent scoping review reported that the median 12-month utilization of chiropractic services was 9.1% (interquartile range (IQR) 6.7–13.1%) globally [5]. The most common reasons for people attending chiropractic care are low back pain, neck pain, and extremity problems [5]. The most common treatments provided by chiropractors include spinal manipulation, soft tissue therapy, and education [5]. Across health systems, chiropractors provide care to a considerable proportion of patients with musculoskeletal conditions [6], playing an important role in their management in the primary care setting.

Chiropractors have diverse views of practice related to the health conditions that they treat. Some chiropractors have an unorthodox view, considered as viewing spinal dysfunctions, termed ‘vertebral subluxations’, as an encumbrance to the expression of health that is corrected to benefit overall patient well-being [7]. This view of practice perceives vertebral subluxations as a negative effect on the body’s innate ability to heal, which can be corrected through spinal manipulation [8–10]. The majority (approximately 70–80%) of chiropractors do not endorse this unorthodox view and provide evidence-based care for the management of musculoskeletal conditions [7, 11–15]. However, previous studies estimated that between 19% to 28% of chiropractors have an unorthodox view of practice [7, 14, 15]. Specifically, McGregor et al. reported that 19% of Canadian chiropractors in 2014 had an unorthodox view of practice [7]. Biggs et al. reported that 28% of Canadian chiropractors in 2002 were considered empiricists, which aligned with an unorthodox perspective and relied on clinical experience as the main method for validating chiropractic [14]. In the United States, McDonald et al. surveyed a random sample of chiropractors in North America in 2004 [15] and found that 5% of chiropractors aligned with an unorthodox view, while 24% reported a middle scope, which tended to combine spinal manipulation of subluxations with other management approaches [15].

It remains unclear to what extent the unorthodox view among some chiropractors influences their patient profiles and treatment approaches. McGregor et al. reported that an unorthodox view of practice among chiropractors was associated with non-evidence-based treatment choices, use of radiographs that was not consistent with evidence-based guidelines, and a negative attitude towards vaccination [7]. Previous literature also suggests that unorthodox views are associated with treatment of non-musculoskeletal conditions [15, 16]. However, studies are needed to assess whether unorthodox views are associated with differences in other patient encounter-level characteristics, including diagnosis, treatment provided, and patient health characteristics. Understanding the patient profiles and treatment approaches of chiropractors with varying views could greatly advance our understanding of chiropractic practice and inform collaboration among chiropractors and other healthcare providers. Describing whether patient profiles and treatment approaches vary by chiropractors with different views on practice provides a more comprehensive perspective of chiropractic care. In turn, understanding these characteristics would guide collaboration and communication between chiropractors and other healthcare providers. This can inform decision-making on referrals or co-management with other healthcare providers, based on a better understanding of the needs and characteristics of chiropractic patients and chiropractic care delivered.

Therefore, the objective of this study was to assess the association between chiropractors’ view of practice (unorthodox versus orthodox) and patient encounter-level characteristics among chiropractors who practice in Ontario, Canada.

## Methods

We conducted a cross-sectional study using data from the Ontario Chiropractic Observation and Analysis STudy (O-COAST) [17]. We reported this study according to the Strengthening the Reporting of Observational Studies in Epidemiology statement [18]. This project has been approved by the Research Ethics Board at the Canadian Memorial Chiropractic College (REB #1404X03).

### Study sample and data source

Eligible for the study were chiropractor participants of O-COAST within the primary care setting in Ontario, Canada. We excluded chiropractor participants with missing data on view of practice (1 chiropractor) and those with data errors (1 chiropractor). In O-COAST, chiropractors were randomly recruited from a list of 3,978 chiropractors registered with the College of Chiropractors of Ontario in 2014. Chiropractors in active clinical practice (full-time, part-time, or locum) in Ontario were eligible to participate. Each chiropractor invited consecutive patients to participate until 100 encounters were recorded per chiropractor, or when four weeks of recording elapsed. A total of 135 randomly selected chiropractors were approached, of which 120 chiropractors were eligible, and 43 agreed to participate (36% response rate). One chiropractor withdrew due to personal reasons and 42 completed the study (98% follow-up rate). The 43 chiropractor participants provided information on 3,523 chiropractor-patient encounters between July 3, 2014 and July 15, 2015. No direct identifiers were collected from the chiropractor. Information on patients was kept confidential by the research team. Re-identification of individuals is not be possible, since only aggregate findings are reported. Additional details on O-COAST methods are available in the published study [17].

Ontario is the largest province by population (~ 14.6 million in 2020) in Canada [19]. A chiropractor in Ontario is a member of a regulated health profession, regulated by the College of Chiropractors of Ontario under the Regulated Health Professions Act, 1991 [20]. Doctors of chiropractic complete a minimum of 7 years of post-secondary education before becoming registered with College of Chiropractors of Ontario [20]. Chiropractic services are not paid through the government-run provincial health insurance plan, which is the Ontario Health Insurance Plan. Fees for chiropractic services may be out-of-pocket or paid by extended health insurance, workers’ compensation for occupational injuries, or automobile insurance for traffic-related injuries.

#### Main independent variable: chiropractors’ view of practice

We defined chiropractors’ view of practice based on a single question with six statements that best describes their predominant view of the conditions treated (Additional File 1) [7]. Responses to the six statements were dichotomized with those viewing vertebral subluxation as an encumbrance to the expression of health that is corrected to benefit patient well-being classified as unorthodox. All other views were classified as orthodox. This question was validated by McGregor et al. [7] and used in studies to define view of practice among chiropractors [21].

#### Dependent variables: patient encounter-level characteristics

We estimated the direction and magnitude of the association between chiropractors’ view of practice and patient encounter-level characteristics. As informed by previous literature [7, 14–16, 22], the following dependent variables were selected a priori:

1. Reason for encounter for a non-musculoskeletal condition (non-musculoskeletal versus other). Informed by previous literature [17], the list of non-musculoskeletal conditions was defined by the authors and included visceral (e.g., digestive, ear, eye, respiratory, skin, urology, circulatory, endocrine and metabolic conditions) and psychological conditions (Additional File 2).
2. Subluxation as diagnosis (subluxation versus other)
3. Duration of patient encounter (continuous variable in minutes)
4. Unimodal manipulative treatment i.e., one intervention only (manual adjustment and/or chiropractic system versus other)
5. In subset of data due to data availability:
  Very good/satisfied health status (i.e., self-rated general health of patient as excellent health/very good, and quality of life as very good, and satisfaction with health as very satisfied/satisfied)
  Activity limitations (none, a few, versus some)

Very good/satisfied health status was defined as a composite measure of three variables, which were self-rated general health, quality of life, and satisfaction with health. Self-rated general health was based on the question, “In general, would you say your health is excellent, very good, good, fair, or poor?” (excellent/very good versus good/fair/poor). Self-rated quality of life was based on the question, “How would you rate your quality of life” (very good versus good/neither poor nor good/poor/very poor). Self-rated satisfaction with life was based on the question, “How satisfied are you with your health?” (very satisfied/satisfied versus neither satisfied nor unsatisfied/fairly dissatisfied/dissatisfied). Activity limitations was based on the question, “how many activities does your pain or discomfort prevent?” (none/a few versus some).

#### Covariates

We included the following patient encounter-level variables as covariates in the models: age (in years); sex (men, women); number of comorbidities (0, 1, 2 or more), and imaging undertaken (yes, no). We identified other potential confounders using the model building approach outlined in the analysis section.

### Analysis

We described the chiropractor participants with respect to sociodemographic, education, and practice-related factors using means [standard deviations (SD)] or medians [interquartile ranges (IQR)] and percentages for continuous and categorical variables, respectively, stratified by chiropractors’ view of practice. Similarly, we described the patient encounters with respect to sociodemographic, treatment- and health-related factors, stratified by chiropractors’ view of practice.

We conducted multilevel logistic regression models to assess the association between chiropractors’ view of practice and the aforementioned patient encounter-level characteristics, accounting for clustering of encounters within chiropractors. The multilevel regression models had two levels (level 1—the patient encounter, level 2—the chiropractor), with level 1 patient encounters nested within level 2 chiropractors. The main independent variable, view of practice, is measured at the chiropractor level, while covariates and outcomes are measured at the patient encounter level. The variance explained by chiropractor level is that explained by both unorthodox/orthodox view of practice and the random intercept. We adjusted for potential confounders in the models, which included confounders determined conceptually and those selected using a model building strategy to assess for a 10% change in the exposure regression coefficient. Variables that led to a 10% change in the exposure regression coefficient were new patient encounter (yes, no), extended health insurance as payment method (yes, no), injury related to motor vehicle collision (yes, no), and injury related to workers’ compensation (yes, no); therefore, these variables were included in the fully adjusted models. We used a link function in the analysis for duration of patient encounter (log-transformed) to account for skewed distributions. The analysis for this study was generated using SAS software, version 9.4. of the SAS System for Windows (Copyright © 2002–2012, SAS Institute Inc., Cary, NC, USA. SAS and all other SAS Institute Inc. product or service names are registered trademarks or trademarks of SAS Institute Inc., Cary, NC, USA.)

We conducted sensitivity analyses to explore the impact of potential misclassification of the chiropractors’ view of practice as described by McGregor et al. [7]. First, we conducted a sensitivity analysis by redefining unorthodox view by those who predominantly view treating vertebral subluxation as unorthodox (i.e., combining those who “treat vertebral subluxation as an encumbrance to the expression of health” and “treat vertebral subluxation as a somatic joint dysfunction and/or related to functional or musculoskeletal problems”) versus all other views defined as orthodox. Second, we dichotomized to chiropractors who predominantly view treating “vertebral subluxations” (i.e., the two aforementioned responses) or “lifestyle and wellness issues”, compared to all other views redefined as orthodox.

## Results

**Fig. 1:**
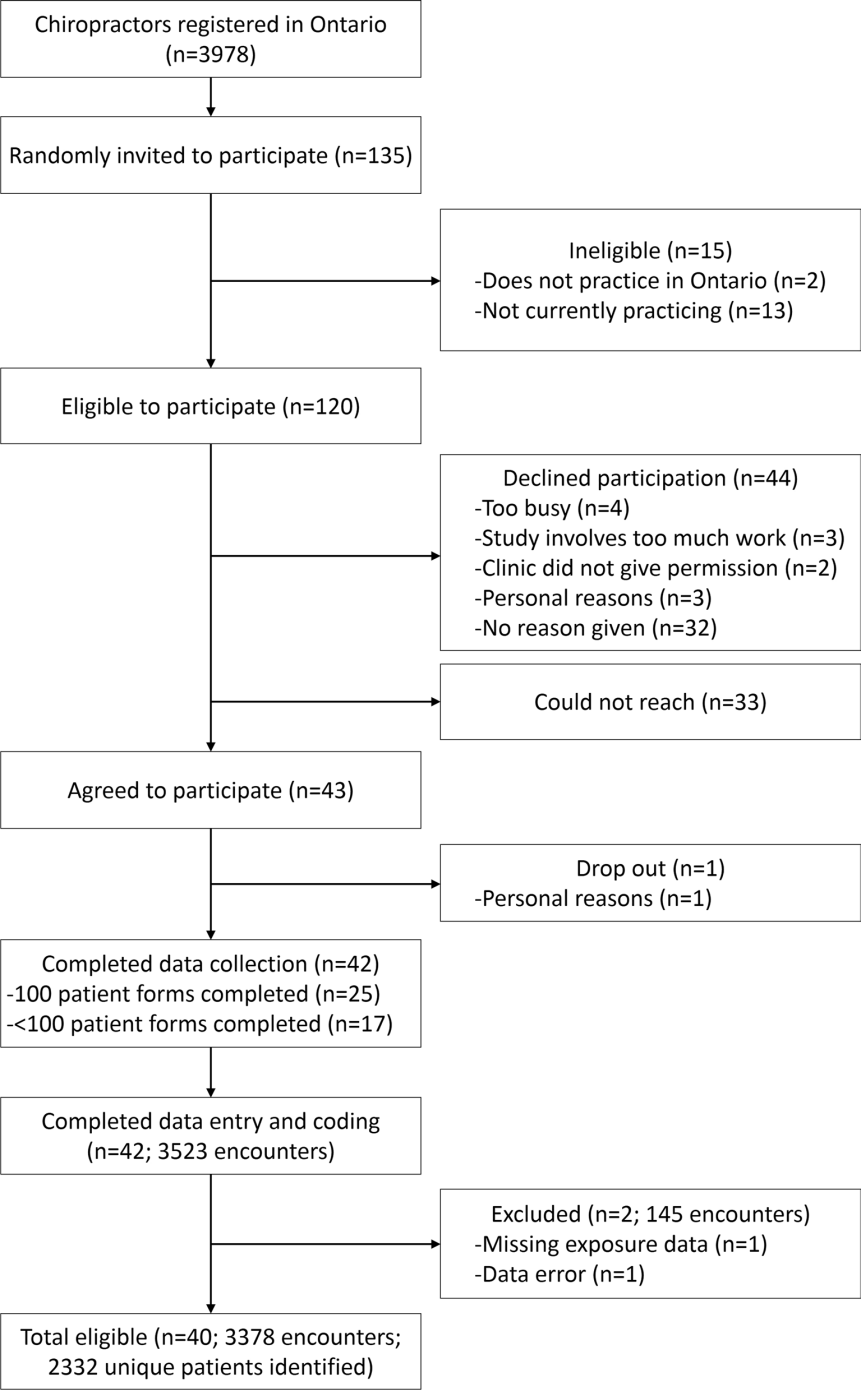
Flow diagram for the enrolment of chiropractors and data collection for the Ontario Chiropractic Observation and Analysis STudy (O-COAST)

The O-COAST data had a total of 42 chiropractor participants and 3,523 chiropractor-patient encounters; we excluded 1 chiropractor due to missing exposure data and 1 chiropractor due to data errors (Fig. 1). Therefore, 40 chiropractor participants and 3378 chiropractor-patient encounters were used for analysis. Of the 3378 encounters, we identified 2332 unique patients (with complete data on date of birth and postal code).

### Chiropractor characteristics

The 40 chiropractors had a mean age of 43.4 years (SD 11.5) and median 12.5 years in practice (IQR 6.0–24.0), with 33% women (Table 1). There were 32 (80%) chiropractors with an orthodox view of practice, and 8 (20%) who had an unorthodox view. Chiropractors with an unorthodox view of practice had a higher number of patient visits per week (median 135.0, IQR 72.5–197.5) than those with orthodox views (median 70.0, IQR 30.0–116.0). A higher proportion of chiropractors with an unorthodox view had imaging services available within the premises of their clinic than chiropractors with orthodox views (50% versus 3%).

**Table 1:** Characteristics of chiropractors participating in O-COAST by view of chiropractic practice^a^ (n = 40)

	All chiropractors (n = 40)	Unorthodox view of practice^a^ (n = 8)	Orthodox view of practice (n = 32)
Chiropractor characteristics^b^			
Women	13 (32.5%)	3 (37.5%)	10 (31.3%)
Age in years, median (IQR)	41.5 (36.0–52.5)	43.5 (39–54.5)	41.0 (34.0–50.5)
Years in practice, median (IQR)	12.5 (6.0–24.0)	13.5 (8.0–28.0)	12.5 (6.0–20.5)
Years since graduation, median (IQR)	14.0 (7.0–27.0)	14.0 (8.5–28.5)	14.0 (7.0–24.5)
Country of graduation			
Canada	33 (82.5%)	7 (87.5%)	26 (81.3%)
USA	6 (15.0%)	0 (0%)	6 (18.8%)
Other	1 (2.5%)	1 (12.5%)	0 (0%)
Holds postgraduate qualification	7 (18.4%)	1 (14.3%)	6 (19.4%)
Practice characteristics^b^			
Number of patient visits per week, median (IQR)	80.0 (32.5–150.0)	135.0 (72.5–197.5)	70.0 (30.0–116.0)
Number of chiropractors at practice			
Solo practitioner	21 (52.5%)	2 (25.0%)	19 (59.4%)
Other chiropractor(s) at practice	19 (47.5%)	6 (75.0%)	13 (40.6%)
Other non-chiropractic healthcare practitioner available at same premises	31 (77.5%)	7 (87.5%)	24 (75.0%)
Imaging services available at same premises	5 (12.0%)	4 (50.0%)	1 (3.1%)
Paper-only clinical records	22 (55.0%)	4 (50.0%)	18 (56.3%)
Type of practice			
General/family	33 (82.5%)	7 (87.5%)	26 (81.3%)
Sports/rehabilitation	5 (12.5%)	1 (12.5%)	5 (15.6%)
Wellness/lifestyle counselling	2 (5.0%)	0 (0%)	1 (3.1%)

*IQR* interquartile range; *O-COAST* Ontario Chiropractic Observation and Analysis STudy

^a^Unorthodox view of practice defined as viewing vertebral subluxation as an encumbrance to the expression of health that is corrected to benefit patient well-being

^b^Number (%) of chiropractors unless otherwise specified

### Patient characteristics

The 2,332 unique patients had a mean age of 48.5 years (SD 18.5), with 58.4% female and 16.2% living in a rural area (Table 2). A lower proportion of patients receiving care from chiropractors with an unorthodox view had extended private health insurance than those seeing chiropractors with orthodox views (3.7% versus 41.6%).

**Table 2:** Characteristics of unique patients in encounters as recorded by chiropractors by view of practice^a^ (n = 2,332)

Patient Characteristics^b^	All patients (n = 2332)	Unorthodox view of practice^a^ (n = 565)	Orthodox view of practice (n = 1767)
Women	1362 (58.4%)	345 (61.1%)	1017 (57.6%)
Age in years, mean (SD)	48.5 (18.5)	47.0 (19.7)	48.9 (18.0)
Age categories, in years^c^			
< 15	101 (4.4%)	33 (5.9%)	68 (3.9%)
15–24	163 (7.0%)	51 (9.2%)	111 (6.3%)
25–44	627 (27.1%)	138 (24.8%)	489 (27.9%)
45–64	966 (41.8%)	216 (38.9%)	750 (42.7%)
65–74	299 (12.9%)	84 (15.1%)	215 (12.3%)
≥ 75	156 (6.8%)	34 (6.1%)	122 (6.9%)
Rural location of residence	378 (16.2%)	98 (17.4%)	280 (15.9%)
Non-English speaking background	93 (4.1%)	25 (4.6%)	68 (3.9%)
Identifies as Aboriginal/Indigenous	2 (0.1%)	0 (0%)	2 (0.1%)
Employment status			
Employed	1377 (64.0%)	316 (62.5%)	1061 (64.5%)
Home duties	102 (4.7%)	51 (2.6%)	89 (5.4%)
Retired	460 (21.4%)	216 (21.2%)	353 (21.5%)
Student	196 (9.1%)	84 (13.2%)	129 (7.8%)
Unemployed/non-employed	17 (0.8%)	3 (0.6%)	14 (0.9%)
*Source of encounter payment*			
Workplace safety and insurance board			
Yes	21 (0.9%)	2 (0.4%)	19 (1.1%)
No	2268 (99.1%)	542 (99.6%)	1726 (98.9%)
Motor vehicle accident^c^			
Yes	65 (2.8%)	9 (1.7%)	56 (3.2%)
No	2224 (97.2%)	535 (98.4%)	1689 (96.8%)
Veterans affairs^c^			
Yes	10 (0.4%)	1 (0.2%)	9 (3.2%)
No	2279 (99.6%)	543 (99.8%)	1736 (96.8%)
Extended private health insurance^c^			
Yes	746 (32.6%)	20 (3.7%)	726 (41.6%)
No	1543 (67.4%)	524 (96.3%)	1019 (58.4%)
Patient paid^c^			
Yes	1567 (68.5%)	418 (76.8%)	1149 (65.9%)
No	722 (31.5%)	126 (23.2%)	596 (34.2%)
No charge^c^			
Yes	59 (2.6%)	25 (4.6%)	34 (1.9%)
No	2230 (97.4%)	519 (95.4%)	1711 (98.1%)
Number of encounters			
1	1964 (84.3%)	519 (91.9%)	1445 (81.9%)
2	260 (11.2%)	37 (6.6%)	223 (12.6%)
≥ 3	105 (4.5%)	9 (1.6%)	96 (5.44%)
*Encounter characteristics*			
Diagnosis			
Subluxation	1096 (32.5%)	545 (74.4%)	551 (20.8%)
Other	2282 (67.6%)	188 (25.7%)	2094 (79.2%)
Duration of encounter in minutes, median (IQR)^c^	15 (10–25)	10 (7–14)	15 (10–30)
Unimodal manipulative treatment	101 (3.0%)	91 (12.4%)	10 (0.4%)
Non-musculoskeletal condition as reason for encounter	30 (0.9%)	22 (3.0%)	8 (0.3%)
Patient characteristics			
Some activity limitations due to pain (n = 1559)	336 (21.6%)	60 (18.2%)	276 (22.4%)
Excellent/very good health status (n = 1559)	1253 (80.4%)	263 (79.9%)	990 (80.5%)

*IQR* interquartile range

^a^Unorthodox view of practice defined as viewing vertebral subluxation as an encumbrance to the expression of health that is corrected to benefit patient well-being

^b^Number (%) of encounters unless otherwise specified

^c^Do not add up to 100% due to missing values

### Clinical encounter characteristics

A higher proportion of patients seeing chiropractors with an unorthodox view had subluxation as a diagnosis (74.4% versus 20.8%) and received unimodal manipulative treatment (12.4% versus 0.4%) than chiropractors with an orthodox view of practice (Table 2). Patients receiving care from chiropractors with an unorthodox view had shorter durations of encounter (median 10 min, IQR 7–14) compared to those with orthodox views (median 15 min, IQR 10–30).

### Association between unorthodox view of practice and patient encounter-level characteristics

Based on fully adjusted analyses, chiropractors with an unorthodox view of practice had higher odds of having patients with a non-musculoskeletal reason for encounter (adjusted odds ratio (aOR) = 16.5, 95% CI 3.2–84.0) and subluxation as diagnosis (aOR = 63.0, 95% CI 4.2–949.1) (Table 3). The encounters of chiropractors with an unorthodox view were 0.6 times shorter than those with orthodox views (95% CI 0.4–0.9). Chiropractor level explained 31.5%, 75.4%, and 48.7% of the variability in non-musculoskeletal reason for encounter, subluxation as diagnosis, and duration of encounter, respectively.

**Table 3:** Effect estimates of the association between unorthodox view of practice^a^ and encounter characteristics (n = 3378 encounters)

	Unadjusted	Age and sex adjusted	Fully adjusted
Subluxation diagnosis^b^			
Unorthodox	OR 108.90 (95% CI 8.46–1401.35)	OR 63.33 (95% CI 4.35–922.72)	OR 63.02 (95% CI 4.18–949.07)
Orthodox	Reference (1.00)	Reference (1.00)	Reference (1.00)
ICC (intercept only: 80.24%)	75.25%	74.98%	75.36%
Duration of encounter^c^			
Unorthodox	0.57 (95% CI 0.38–0.84)	0.57 (95% CI 0.38–0.84)	0.59 (95% CI 0.41–0.86)
Orthodox	Reference (0.00)	Reference (0.00)	Reference (0.00)
ICC (intercept only: 55.10%)	50.67%	50.70%	48.67%
Unimodal manipulative treatment^d^			
Unorthodox	OR 6.51 (95% CI 0.24–179.77)	OR 7.73 (95% CI 0.24–247.97)	OR 7.37 (95% CI 0.22–243.55)
Orthodox	Reference (1.00)	Reference (1.00)	Reference (1.00)
ICC (intercept only: 71.63%)	72.19%	73.01%	72.63%
Non-musculoskeletal condition as reason for encounter^e^			
Unorthodox	OR 9.66 (2.92–31.92)	OR 9.93 (95% CI 2.52–39.20)	OR 16.46 (95% CI 3.23–83.96)
Orthodox	Reference (1.00)	Reference (1.00)	Reference (1.00)
ICC (intercept only: 33.66%)	23.09%	27.19%	31.45%

*CI* confidence interval, *ICC* intraclass correlation coefficientl, *OR* odds ratio

^a^Unorthodox view of practice defined as viewing vertebral subluxation as an encumbrance to the expression of health that is corrected to benefit patient well-being; all other views of practice considered orthodox

^b^Refers to diagnosis that used the term “subluxation”; model adjusted for age, sex, new patient encounter, extended health insurance as payment, injury related to motor vehicle collision, injury related to workers’ compensation, and imaging ordered during encounter

^c^Refers to duration of patient encounter in minutes (229 encounters excluded due to missing or nonsensical data); based on linear (log-transformed) regression models adjusted for age, sex, new patient encounter, extended health insurance as payment method, injury related to motor vehicle collision, injury related to workers’ compensation, and imaging ordered during encounter

^d^Refers to treatment that consisted of manual adjustments or treatment using a chiropractic system only; model adjusted for age, sex, new patient encounter, and extended health insurance as payment method *(other variables could not be included because model would not converge)*

^e^Refers to reason for encounter for a non-musculoskeletal condition; model adjusted for age, sex, new patient encounter, and extended health insurance as payment method *(other variables could not be included because model would not converge)*

We observed no association between unorthodox view and unimodal manipulative treatment (Table 3) or patient health characteristics (i.e., some activity limitations or excellent/very good health status) based on fully adjusted analyses (Table 4). Chiropractor level explained 10.0% and 7.7% of the variability in some activity limitations and excellent/very good health status, respectively.

**Table 4:** Odds ratio of the association between unorthodox view of practice^a^ and patient health characteristics (n = 1559)

	Odds ratio (95% confidence interval)		
	Unadjusted	Age and sex adjusted	Fully adjusted
Some activity limitations due to pain^b^			
Unorthodox	0.77 (0.44–1.36)	0.77 (0.42–1.39)	0.76 (0.41–1.42)
Orthodox	Reference (1.00)	Reference (1.00)	Reference (1.00)
ICC (intercept only: 8.68%)	8.74%	9.37%	10.01%
Excellent/very good health status^c^			
Unorthodox	0.95 (0.56–1.62)	0.91 (0.53–1.58)	0.85 (0.49–1.49)
Orthodox	Reference (1.00)	Reference (1.00)	Reference (1.00)
ICC (intercept only: 7.25%)	7.65%	7.93%	7.74%

*ICC* intraclass correlation coefficient

^a^Unorthodox view of practice defined as viewing vertebral subluxation as an encumbrance to the expression of health that is corrected to benefit patient well-being; all other views of practice considered orthodox

^b^Refers to some activities prevented by pain or discomfort; model adjusted for age, sex, new patient encounter, extended health insurance as payment, injury related to motor vehicle collision, injury related to workers’ compensation, and imaging ordered during encounter

^c^Refers to self-rated general health of patient as excellent health/very good, quality of life as very good, and satisfaction with health as very satisfied/satisfied; model adjusted for age, sex, new patient encounter, extended health insurance as payment, injury related to motor vehicle collision, injury related to workers’ compensation, and imaging ordered during encounter

### Sensitivity analyses

#### Unorthodox view when defined as predominantly treating subluxations (combining two response options)

Based on fully adjusted analyses, association between unorthodox view and non-musculoskeletal reason for encounter, subluxation as diagnosis, or duration of encounter was attenuated, but the association remained (Additional File 3a–d). Chiropractor level explained a high proportion of the variability in these outcomes. We observed no association between unorthodox view and unimodal manipulative treatment or patient health characteristics, similar to primary analyses.

#### Unorthodox view when defined as predominantly treating subluxations or lifestyle and wellness issues

Association between unorthodox view and non-musculoskeletal reason-for-encounter or duration of encounter was attenuated, but the association remained based on fully adjusted analyses (Additional File 4a–d). Chiropractor level explained a high proportion of the variability in these outcomes. The association between unorthodox view and subluxation as diagnosis was greater compared to primary analyses (aOR 96.2, 95% CI 14.2–650.8); chiropractor explained 69.4% of the variability in the outcome. We could not assess unimodal manipulative treatment due to small numbers. Similar to primary analyses, we found no association between unorthodox view and patient health characteristics.

## Discussion

We found that 80% of Ontario chiropractors in our study had an orthodox view of practice whereas 20% of chiropractors had an unorthodox view. Chiropractors with an unorthodox view were associated with treating a non-musculoskeletal reason for encounter and subluxation as diagnosis. Encounters of chiropractors with an unorthodox view were shorter than encounters of those with orthodox views. In the multilevel models, chiropractor level explained a high proportion of the variability in non-musculoskeletal reason for encounter, subluxation as diagnosis, and encounter duration. There was no association between unorthodox view of practice and unimodal manipulative treatment or patient health characteristics.

Our findings that 20% of chiropractors in Ontario had an unorthodox view is similar to previous studies conducted in Canada and elsewhere. When we reclassified chiropractors to include those predominantly treating subluxations as having an unorthodox view in our sensitivity analysis, we found that 30% of chiropractors would have an unorthodox view; however, the associations remained similar. Previous literature reported that 19% to 28% of chiropractors in Canada [7, 14] had unorthodox views of practice, and 5% to 24% of chiropractors in North America had a focused (unorthodox view) or middle scope (subluxation adjusting combined with other procedures) [15]. Thus, some of these differences may be owing to varied definitions (e.g., focused and middle scope) and that the study surveyed a random sample of chiropractors more broadly in North America in 2004.

Our study results advances the knowledge on patient profiles and treatment approaches among chiropractors with varying views of practice. In their cross-sectional study of a random sample of Canadian chiropractors in 2010, McGregor et al. reported that unorthodox view of practice was associated with non-evidence-based treatment choices (OR 4.2, 95% CI 2.2–8.0) and non-guidelines-based radiograph use (OR 3.0, 95% CI 1.7–5.4) [7]. In the study by McGregor et al., non-evidence-based treatment choices included treating allergies, attention deficit hyperactivity disorder, diabetes, multiple sclerosis, cancer, and cystic fibrosis [7]. In our study, we found an association between an unorthodox view of practice and non-musculoskeletal conditions as the reason for encounter. We included visceral (e.g., cancer, immune, endocrine, metabolic, nutritional, cardiovascular, respiratory, gastrointestinal, urinary conditions) and psychological conditions in the list of non-musculoskeletal conditions. Our findings fill an important knowledge gap by providing new evidence on the association between unorthodox view of practice and subluxation as diagnosis and duration of encounter, after accounting for a wide range of potential confounders.

Findings have implications for understanding chiropractic practice and informing shared decision-making between patients and chiropractors. Our study more comprehensively describes the characteristics of patients treated by chiropractors with an unorthodox view and their associated diagnosis and encounter characteristics. This can help guide patient expectations when making informed decisions about their treatment options when seeking care from chiropractors. For example, understanding the type of care provided by chiropractors can facilitate shared decision-making with patients, including enabling patients to make reasoned informed choices and guiding communication on appropriate choices of treatment [23]. Although non-musculoskeletal conditions comprise a small proportion (about 3%) of patients receiving chiropractic care [17], we found that chiropractors’ unorthodox view was associated with treating non-musculoskeletal conditions. This highlights a potential area for education to guide evidence-based approaches in the management of non-musculoskeletal conditions [24], as well as the understanding that people with musculoskeletal conditions may have non-musculoskeletal comorbidities that impact overall health and well-being [25].

Our study results can also inform interprofessional collaboration between chiropractors and healthcare providers. Previous studies reported that orthopedic surgeons and obstetricians in Canada considered diverse views of practice among chiropractors as a barrier to interprofessional collaboration [26–28]. This included issues such as scope of practice associated with the treatment of non-musculoskeletal conditions and non-evidence-based care from chiropractors, as well as the unintended consequence of stigmatization of the profession impacting trustworthiness, collaborative opportunities, and professional identity [29]. Our study found that 20% of chiropractors had an unorthodox view of practice. Ongoing communication related to diagnosis and types of conditions treated may help to increase collaboration between chiropractors and other healthcare providers to improve patient outcomes. As defined by the World Health Organization, “collaborative practice happens when multiple health workers from different professional backgrounds work together with patients, families, carers, and communities to deliver the highest quality of care across settings” [30]. Describing patient profiles can improve coordinated care between chiropractors and other healthcare providers that is responsive to the needs of the population. We found that an unorthodox view of practice is associated with treating patients with non-musculoskeletal conditions, and care for these patients may include appropriate referrals or co-management with other providers. Identifying treatment approaches among chiropractors can improve access to health interventions and guide appropriate and timely referral that matches the provider’s expertise to the needs of patients. Moreover, understanding patient and treatment characteristics can facilitate effective communication, enhanced by team members talking and actively listening while recognizing each other’s body of knowledge [30].

### Strengths and limitations

The study has a number of strengths. First, O-COAST data used a number of approaches to minimize measurement error. O-COAST used a valid and reliable method of recording patient encounters in the primary care setting [31]. Data collection forms were modified and pilot tested to be relevant for chiropractic practice in Ontario. The O-COAST data were collected prospectively, including patient encounter-level characteristics recorded during patient encounters, eliminating potential error with recall or approaches involving chart reviews. Second, we used a validated survey question to define chiropractors’ view of practice, which has been used in previous studies [7, 21]. We also conducted sensitivity analyses to explore potential misclassification of unorthodox views, which found similar associations to the primary analysis. Third, reasons for encounters and diagnoses were classified based on a validated coding system [32, 33] and involved a quality assurance protocol to ensure reliability across trained coders. Finally, we adjusted for potential confounders and clustering of encounters within chiropractors in the analysis.

The study has limitations. First, despite inviting a random sample of chiropractors to participate, O-COAST had a 36% response rate and has potential selection bias. This response rate is higher than similar studies assessing patient encounters in general practice (27% response rate) [34] and chiropractic practice (33% response rate) [35] in Australia. Second, this is a cross-sectional study, so associations between chiropractors’ views of practice and patient health characteristics are based on one time point only. There is also potential for residual confounding; future longitudinal studies in this area that account for a wide range of confounders are needed. Third, the small numbers of encounters for specific non-musculoskeletal conditions within certain categories precluded us from examining them in meaningful ways, and is unlikely to affect our study results. In O-COAST, the proportion of encounters for neurological problems was 0.93% (95% CI 0.5–1.8), all types of headaches was 0.68% (95% CI 0.3–1.4), and concussion was 0.48% (95% CI 0.2–1.3) [17]. Finally, some study results had wide 95% confidence intervals due to the smaller number of chiropractor participants and that chiropractor level explained a high proportion of the variability in outcomes.

## Conclusions

Chiropractors with an unorthodox view of practice were associated with treating a non-musculoskeletal reason for encounter, subluxation as diagnosis, and shorter duration of encounter. Chiropractor level explained a high proportion of the variability in these outcomes. Findings have implications for understanding chiropractic practice and informing interprofessional collaboration and future research. Describing the patient profiles and treatment approaches of chiropractors based on view of practice can help guide patient expectations and communication when making informed decisions about their treatment options through shared decision-making. Our findings highlight a potential area for education among chiropractors to guide evidence-based approaches for non-musculoskeletal conditions. In addition, communication related to diagnosis and types of conditions treated may facilitate collaboration between chiropractors and other healthcare providers to improve patient outcomes.

## Supplementary Information

**Additional file 1.** Question assessing chiropractors’ view of practice based on study by McGregor et al.

**Additional file 2.** List of non-musculoskeletal conditions (including visceral and psychological conditions).

**Additional file 3.Additional file 3a.** Characteristics of chiropractors participating in Ontario Chiropractic Observation and Analysis STudy (O-COAST) by view of chiropractic practice (with unorthodox view classified as predominantly treating subluxations) (n = 40)^a^. **Additional File 3b.** Characteristics of unique patients in encounters as recorded by participating chiropractors by view of practice (with unorthodox view classified as predominantly treating subluxations) (n = 2332)^a^. **Additional File 3c.** Effect estimates of the association between unorthodox view of practicea and encounter characteristics based on unadjusted, age and sex adjusted, and fully adjusted models in sensitivity analysis (n = 3378 encounters). **Additional File 3d.** Odds ratio of the association between unorthodox view of practicea and patient health characteristics based on unadjusted, age and sex adjusted, and fully adjusted models in sensitivity analysis (n = 1559).

**Additional file 4.Additional File 4a.** Characteristics of chiropractors participating in Ontario Chiropractic Observation and Analysis STudy (O-COAST) by view of chiropractic practice (with unorthodox view classified as predominantly treating subluxation or lifestyle/wellness issues) (n = 40)^a^. **Additional File 4b.** Characteristics of unique patients in encounters as recorded by participating chiropractors by view of practice (with unorthodox view classified as predominantly treating subluxation or lifestyle/wellness issues) (n = 2332)^a^. **Additional File 4c.** Effect estimates of the association between unorthodox view of practicea and encounter characteristics based on unadjusted, age and sex adjusted, and fully adjusted models in sensitivity analysis (n = 3378 encounters). **Additional File 4d.** Odds ratio of the association between unorthodox view of practicea and patient health characteristics based on unadjusted, age and sex adjusted, and fully adjusted models in sensitivity analysis (n = 1559).

## Data Availability

The datasets generated and/or analysed during the current study are not publicly available due to privacy restrictions but are available from the corresponding author on reasonable request.

## Abbreviations

aOR: Adjusted odds ratio
CI: Confidence interval
IQR: Interquartile range
O-COAST: Ontario Chiropractic Observation and Analysis STudy
OR: Odds ratio
SD: Standard deviation
