# Supplementary material for: The association between chiropractors’ view of practice and patient encounter-level characteristics in Ontario, Canada: a cross-sectional study
Source: Chiropr Man Therap. 2021 Sep 28;29:41. doi: 10.1186/s12998-021-00398-x (PMC8477501; doi:10.1186/s12998-021-00398-x)
Supplement: Supplementary file 1 — Additional file 1. Question assessing chiropractors’ view of practice based on study by McGregor et al. [file 12998_2021_398_MOESM1_ESM.docx]

**SUPPLEMENTAL FILE**

**Title:** The association between chiropractors’ view of practice and patient encounter-level characteristics in Ontario, Canada: A cross-sectional study

**Authors:** Jessica J. Wong, DC, MPH; Sheilah Hogg-Johnson, PhD; André E. Bussières, DC, PhD; Simon D. French, PhD, MPH, BAppSc(Chiropractic); Silvano A. Mior, DC, PhD

**Corresponding author:** Jessica J. Wong, Centre for Disability Prevention and Rehabilitation, 2000 Simcoe Street North, Oshawa, Ontario L1G 0C5 Canada; Email: [jessica.wong@ontariotechu.ca](mailto:jessica.wong@ontariotechu.ca)

**Contents of Additional Files:**

- **Additional File 1.** Question assessing chiropractors’ view of practice based on study by McGregor et al
- **Additional File 2.** List of non-musculoskeletal conditions (including visceral and psychological conditions)
- **Additional File 3a.** Characteristics of chiropractors participating in Ontario Chiropractic Observation and Analysis STudy (O-COAST) by view of chiropractic practice (with unorthodox view classified as predominantly treating subluxations) (n=40)^a^
- **Additional File 3b.** Characteristics of unique patients in encounters as recorded by participating chiropractors by view of practice (with unorthodox view classified as predominantly treating subluxations) (n=2,332)^a^
- **Additional File 3c.** Effect estimates of the association between unorthodox view of practice^a^ and encounter characteristics based on unadjusted, age and sex adjusted, and fully adjusted models in sensitivity analysis (n=3378 encounters)
- **Additional File 3d.** Odds ratio of the association between unorthodox view of practice^a^ and patient health characteristics based on unadjusted, age and sex adjusted, and fully adjusted models in sensitivity analysis (n=1559)
- **Additional File 4a.** Characteristics of chiropractors participating in Ontario Chiropractic Observation and Analysis STudy (O-COAST) by view of chiropractic practice (with unorthodox view classified as predominantly treating subluxation or lifestyle/wellness issues) (n=40)^a^
- **Additional File 4b.** Characteristics of unique patients in encounters as recorded by participating chiropractors by view of practice (with unorthodox view classified as predominantly treating subluxation or lifestyle/wellness issues) (n=2,332)^a^
- **Additional File 4c.** Effect estimates of the association between unorthodox view of practice^a^ and encounter characteristics based on unadjusted, age and sex adjusted, and fully adjusted models in sensitivity analysis (n=3378 encounters)
- **Additional File 4d.** Odds ratio of the association between unorthodox view of practice^a^ and patient health characteristics based on unadjusted, age and sex adjusted, and fully adjusted models in sensitivity analysis (n=1559)

**Additional File 1.** Question assessing chiropractors’ view of practice based on study by McGregor et al^1^

Which ONE of the following best describes the predominant view you have of the conditions you treat?

- I treat the broadest spectrum of health concerns and may include lifestyle and wellness issues
- I treat musculoskeletal or neuromusculoskeletal problems and may include specific disorders such as low back and neck-related pain
- I treat a combination of general problems and biomechanical groups
- I treat a combination of biomechanical groups and organ/visceral complaints
- I treat vertebral subluxation as a somatic joint dysfunction and/or related to functional or musculoskeletal problems
- I treat vertebral subluxation as an encumbrance to the expression of health – vertebral subluxation is seen as an entity in and of itself, which is corrected to benefit patient well-being
