# Supplementary material for: The association between chiropractors’ view of practice and patient encounter-level characteristics in Ontario, Canada: a cross-sectional study
Source: Chiropr Man Therap. 2021 Sep 28;29:41. doi: 10.1186/s12998-021-00398-x (PMC8477501; doi:10.1186/s12998-021-00398-x)
Supplement: Supplementary file 2 — Additional file 2. List of non-musculoskeletal conditions (including visceral and psychological conditions). [file 12998_2021_398_MOESM2_ESM.docx]

**Additional File 2.** List of non-musculoskeletal conditions (including visceral and psychological conditions)

- Allergy
- Asthma
- Attention deficit disorders
- Bedwetting
- Cancer
- Delayed development
- Ear problems and infections
- Endocrine, metabolic and nutritional conditions
- Eye
- Feeding problem
- Gastrointestinal conditions
- High blood pressure
- Immune complaint
- Irritable infant and infantile colic
- Multiple sclerosis
- Plagiocephaly
- Psychological
- Respiratory conditions
- Skin
- Sleep problem
- Speech issue
- Teething
- Testicular pain
- Urinary conditions
