# Supplementary material for: The association between chiropractors’ view of practice and patient encounter-level characteristics in Ontario, Canada: a cross-sectional study
Source: Chiropr Man Therap. 2021 Sep 28;29:41. doi: 10.1186/s12998-021-00398-x (PMC8477501; doi:10.1186/s12998-021-00398-x)
Supplement: Supplementary file 3 — Additional file 3.Additional file 3a. Characteristics of chiropractors participating in Ontario Chiropractic Observation and Analysis STudy (O-COAST) by view of chiropractic practice (with unorthodox view classified as predominantly treating subluxations) (n = 40)a. Additional File 3b. Characteristics of unique patients in encounters as recorded by participating chiropractors by view of practice (with unorthodox view classified as predominantly treating subluxations) (n = 2332)a. Additional File 3c. Effect estimates of the association between unorthodox view of practicea and encounter characteristics based on unadjusted, age and sex adjusted, and fully adjusted models in sensitivity analysis (n = 3378 encounters). Additional File 3d. Odds ratio of the association between unorthodox view of practicea and patient health characteristics based on unadjusted, age and sex adjusted, and fully adjusted models in sensitivity analysis (n = 1559). [file 12998_2021_398_MOESM3_ESM.docx]

**Additional File 3a.** Characteristics of chiropractors participating in Ontario Chiropractic Observation and Analysis STudy (O-COAST) by view of chiropractic practice (with unorthodox view classified as predominantly treating subluxations) (n=40)^a^

|  | **All chiropractors (n=40)** | **Unorthodox view of practice^a^ (n=12)** | **Orthodox view of practice (n=28)** |
| --- | --- | --- | --- |
| **Chiropractor characteristics^b^** |  |  |  |
| Women | 13 (32.5%) | 6 (50.0%) | 7 (25.0%) |
| Age in years, median (IQR) | 41.5 (36.0-52.5) | 41.5 (38.5-49.0) | 41.5 (31.5-53.0) |
| Years in practice, median (IQR) | 12.5 (6.0-24.0) | 12.5 (7.0-22.0) | 13.0 (6.0-24.0) |
| Years since graduation,  median (IQR) | 14.0 (7.0-27.0) | 13.0 (8.0-22.0) | 15.0 (7.0-27.0) |
| **Country of graduation** |  |  |  |
| Canada | 33 (82.5%) | 9 (75.0%) | 24 (85.7%) |
| USA | 6 (15.0%) | 2 (16.7%) | 4 (14.3%) |
| Other | 1 (2.5%) | 1 (8.3%) | 0 (0%) |
| Holds postgraduate qualification | 7 (18.4%) | 1 (9.1%) | 6 (22.2%) |
| **Practice characteristics^b^** |  |  |  |
| Number of patient visits per week, median (IQR) | 80.0 (32.5-150.0) | 72.5 (30.0-162.5) | 80.0 (35.0-137.5) |
| **Number of chiropractors at practice** |  |  |  |
| Solo practitioner | 21 (52.5%) | 5 (41.7%) | 16 (57.1%) |
| Other chiropractor(s) at practice | 19 (47.5%) | 7 (58.3%) | 12 (42.9%) |
| Other non-chiropractic healthcare  practitioner available at same premises | 31 (77.5%) | 10 (83.3%) | 21 (75.0%) |
| Imaging services available at same premises | 5 (12.0%) | 4 (33.3%) | 1 (3.6%) |
| Paper-only clinical records | 22 (55.0%) | 7 (58.3%) | 15 (53.6%) |
| **Type of practice** |  |  |  |
| General/family | 33 (82.5%) | 11 (91.7%) | 22 (78.6%) |
| Sports/rehabilitation | 5 (12.5%) | 0 (0%) | 5 (17.9%) |
| Wellness/lifestyle counselling | 2 (5.0%) | 1 (8.3%) | 1 (3.6%) |

IQR – interquartile range

^a^Unorthodox view of practice defined as predominantly treating vertebral subluxations (all other views classified as orthodox)

^b^Number (%) of chiropractors unless otherwise specified

**Additional File 3b.** Characteristics of unique patients in encounters as recorded by participating chiropractors by view of practice (with unorthodox view classified as predominantly treating subluxations) (n=2,332)^a^

| **Patient Characteristics^b^** | **All chiropractors (n=2,332)** | **Unorthodox view of practice^a^ (n=698)** | **Orthodox view of practice (n=1,634)** |
| --- | --- | --- | --- |
| Women | 1362 (58.4%) | 429 (61.5%) | 933 (57.1%) |
| Age in years, mean (SD) | 48.5 (18.5) | 47.5 (19.2) | 48.8 (18.1) |
| **Age categories, in years^c^** |  |  |  |
| <15 | 101 (4.4%) | 35 (5.1%) | 66 (4.1%) |
| 15–24 | 163 (7.0%) | 61 (8.9%) | 101 (6.2%) |
| 25–44 | 627 (27.1%) | 175 (25.5%) | 452 (27.8%) |
| 45–64 | 966 (41.8%) | 269 (39.2%) | 697 (42.9%) |
| 65–74 | 299 (12.9%) | 105 (15.3%) | 194 (12.0%) |
| ≥75 | 156 (6.8%) | 42 (6.1%) | 114 (7.0%) |
| Rural location of residence | 378 (16.2%) | 100 (14.3%) | 278 (17.0%) |
| Non-English speaking background | 93 (4.1%) | 32 (4.7%) | 61 (3.8%) |
| Identifies as Aboriginal/Indigenous | 2 (0.1%) | 1 (0.2%) | 1 (0.1%) |
| **Employment status** |  |  |  |
| Employed | 1377 (64.0%) | 402 (63.6%) | 975 (64.1%) |
| Home duties | 102 (4.7%) | 21 (3.3%) | 81 (5.3%) |
| Retired | 460 (21.4%) | 128 (20.3%) | 332 (21.8%) |
| Student | 196 (9.1%) | 77 (12.2%) | 119 (7.8%) |
| Unemployed/non-employed | 17 (0.8%) | 4 (0.6%) | 13 (0.9%) |
| **Source of encounter payment** |  |  |  |
| **Workplace safety and insurance board** |  |  |  |
| Yes | 21 (0.9%) | 2 (0.3%) | 19 (1.2%) |
| No | 2268 (99.1%) | 674 (99.7%) | 1594 (98.9%) |
| **Motor vehicle accident^c^** |  |  |  |
| Yes | 65 (2.8%) | 10 (1.5%) | 55 (3.4%) |
| No | 2224 (97.2%) | 666 (98.5%) | 1558 (96.6%) |
| **Veterans affairs^c^** |  |  |  |
| Yes | 10 (0.4%) | 1 (0.2%) | 9 (0.6%) |
| No | 2279 (99.6%) | 675 (99.9%) | 1558 (96.6%) |
| **Extended private health insurance^c^** |  |  |  |
| Yes | 746 (32.6%) | 83 (12.3%) | 663 (41.1%) |
| No | 1543 (67.4%) | 593 (87.7%) | 950 (58.9%) |
| **Patient paid^c^** |  |  |  |
| Yes | 1567 (68.5%) | 497 (73.5%) | 1070 (66.3%) |
| No | 722 (31.5%) | 179 (26.5%) | 543 (33.7%) |
| **No charge^c^** |  |  |  |
| Yes | 59 (2.6%) | 29 (4.3%) | 30 (1.9%) |
| No | 2230 (97.4%) | 647 (95.7%) | 1604 (99.4%) |
| **Number of encounters** |  |  |  |
| 1 | 1964 (84.3%) | 604 (86.7%) | 1360 (83.3%) |
| 2 | 260 (11.2%) | 62 (8.9%) | 198 (12.1%) |
| ≥3 | 105 (4.5%) | 31 (4.5%) | 74 (4.5%) |
| **Encounter characteristics** |  |  |  |
| **Diagnosis** |  |  |  |
| Subluxation | 1096 (32.5%) | 619 (63.0%) | 477 (19.9%) |
| Other | 2282 (67.6%) | 363 (37.0%) | 1919 (80.1%) |
| Duration of encounter in minutes, median (IQR)^c^ | 15 (10-25) | 11.0 (8.0-15.0) | 15.0 (10.0-30.0) |
| Unimodal treatment | 101 (3.0%) | 91 (9.3%) | 10 (0.4%) |
| Non-musculoskeletal condition as reason for encounter | 30 (0.9%) | 22 (2.2%) | 8 (0.3%) |
| **Patient characteristics** |  |  |  |
| Some activity limitations due to pain (n=1559) | 336 (21.6%) | 74 (16.4%) | 262 (23.7%) |
| Excellent/very good health status (n=1559) | 1253 (80.4%) | 363 (80.5%) | 890 (80.3%) |

IQR – interquartile range

^a^Unorthodox view of practice defined as predominantly treating vertebral subluxations (all other views classified as orthodox)

^b^Number (%) of encounters unless otherwise specified

^c^Do not add up to 100% due to missing values

**Additional File 3c.** Effect estimates of the association between unorthodox view of practice^a^ and encounter characteristics based on unadjusted, age and sex adjusted, and fully adjusted models in sensitivity analysis (n=3378 encounters)

|  | **Unadjusted** | **Age and Sex Adjusted** | **Fully Adjusted** |
| --- | --- | --- | --- |
| **Subluxation diagnosis^b^** |  |  |  |
| Unorthodox | OR 52.85 (5.36-521.14) | OR 32.57 (3.16-335.58) | 32.62 (3.09-344.49) |
| Orthodox | Reference (1.00) | Reference (1.00) | Reference (1.00) |
| ICC (intercept only: 80.24%) | 75.94% | 75.36% | 75.70% |
| **Duration of encounter^c^** |  |  |  |
| Unorthodox | 0.67 (0.47-0.94) | 0.66 (0.45-0.94) | 0.68 (95% CI 0.49-0.94) |
| Orthodox | Reference (0.00) | Reference (0.00) | Reference (0.00) |
| ICC (intercept only: 55.10%) | 52.33% | 52.18% | 50.01% |
| **Unimodal treatment^d^** |  |  |  |
| Unorthodox | 3.34 (0.15-76.76) | 3.68 (0.14-94.20) | 3.49 (0.14-90.01) |
| Orthodox | Reference (1.00) | Reference (1.00) | Reference (1.00) |
| ICC (intercept only: 71.63%) | 71.99% | 72.75% | 72.37% |
| **Non-musculoskeletal condition as reason for encounter^e^** |  |  |  |
| Unorthodox | 5.79 (1.71-19.59) | 6.13 (1.56-23.99) | 7.44 (1.58-35.05) |
| Orthodox | Reference (1.00) | Reference (1.00) | Reference (1.00) |
| ICC (intercept only: 33.66%) | 26.92% | 29.54% | 34.90% |

CI – confidence interval; ICC – intraclass correlation coefficient; OR – odds ratio

^a^Unorthodox view of practice defined as those who predominantly view treating vertebral subluxation as unorthodox; all other views of practice considered orthodox

^b^Refers to diagnosis that used the term “subluxation”; model adjusted for age, sex, new patient encounter, extended health insurance as payment, injury related to motor vehicle collision, injury related to workers’ compensation, and imaging ordered during encounter

^c^Refers to duration of patient encounter in minutes (229 encounters excluded due to missing or nonsensical data); based on linear (log-transformed) regression models adjusted for age, sex, new patient encounter, extended health insurance as payment method, injury related to motor vehicle collision, injury related to workers’ compensation, and imaging ordered during encounter

^d^Refers to treatment that consisted of manual adjustments or treatment using a chiropractic system only; model adjusted for age, sex, new patient encounter, and extended health insurance as payment method *(other variables could not be included because model would not converge)*

^e^Refers to reason for encounter for a non-musculoskeletal condition; model adjusted for age, sex, new patient encounter, and extended health insurance as payment method *(other variables could not be included because model would not converge)*

**Additional File 3d.** Odds ratio of the association between unorthodox view of practice^a^ and patient health characteristics based on unadjusted, age and sex adjusted, and fully adjusted models in sensitivity analysis (n=1559)

|  | **Odds Ratio (95% Confidence Interval)** | | |
| --- | --- | --- | --- |
|  | **Unadjusted** | **Age and Sex Adjusted** | **Fully Adjusted** |
| **Some activity limitations due to pain^b^** |  |  |  |
| Unorthodox | 0.62 (0.38-1.01) | 0.61 (0.37-1.02) | 0.60 (0.35-1.02) |
| Orthodox | Reference (1.00) | Reference (1.00) | Reference (1.00) |
| ICC (intercept only: 8.68%) | 7.98% | 8.56% | 9.09% |
| **Excellent/very good health status^c^** |  |  |  |
| Unorthodox | 1.04 (0.65-1.67) | 1.10 (0.67-1.78) | 1.01 (0.62-1.66) |
| Orthodox | Reference (1.00) | Reference (1.00) | Reference (1.00) |
| ICC (intercept only: 7.25%) | 7.63% | 7.86% | 7.84% |

ICC - intraclass correlation coefficient

^a^Unorthodox view of practice defined as those who predominantly view treating vertebral subluxation as unorthodox; all other views of practice considered orthodox

^b^Refers to some activities prevented by pain or discomfort; model adjusted for age, sex, new patient encounter, extended health insurance as payment, injury related to motor vehicle collision, injury related to workers’ compensation, and imaging ordered during encounter

^c^Refers to self-rated general health of patient as excellent health/very good, quality of life as very good, and satisfaction with health as very satisfied/satisfied; model adjusted for age, sex, new patient encounter, extended health insurance as payment, injury related to motor vehicle collision, injury related to workers’ compensation, and imaging ordered during encounter
